# Human Ageing Genomic Resources: new and updated databases

**DOI:** 10.1093/nar/gkx1042

**Published:** 2017-11-07

**Authors:** Robi Tacutu, Daniel Thornton, Emily Johnson, Arie Budovsky, Diogo Barardo, Thomas Craig, Eugene Diana, Gilad Lehmann, Dmitri Toren, Jingwei Wang, Vadim E Fraifeld, João P de Magalhães

**Affiliations:** Integrative Genomics of Ageing Group, Institute of Ageing and Chronic Disease, University of Liverpool, Liverpool L7 8TX, UK; Computational Biology of Aging Group, Institute of Biochemistry, Romanian Academy, Bucharest 060031, Romania; The Shraga Segal Department of Microbiology, Immunology and Genetics, Center for Multidisciplinary Research on Aging, Ben-Gurion University of the Negev, Beer-Sheva 84105, Israel; Judea Regional Research & Development Center, Carmel 90404, Israel; Department of Biochemistry, Yong Loo Lin School of Medicine, National University of Singapore, Singapore City 117597, Singapore; Science Division, Yale-NUS College, Singapore City 138527, Singapore

## Abstract

In spite of a growing body of research and data, human ageing remains a poorly understood process. Over 10 years ago we developed the Human Ageing Genomic Resources (HAGR), a collection of databases and tools for studying the biology and genetics of ageing. Here, we present HAGR’s main functionalities, highlighting new additions and improvements. HAGR consists of six core databases: (i) the GenAge database of ageing-related genes, in turn composed of a dataset of >300 human ageing-related genes and a dataset with >2000 genes associated with ageing or longevity in model organisms; (ii) the AnAge database of animal ageing and longevity, featuring >4000 species; (iii) the GenDR database with >200 genes associated with the life-extending effects of dietary restriction; (iv) the LongevityMap database of human genetic association studies of longevity with >500 entries; (v) the DrugAge database with >400 ageing or longevity-associated drugs or compounds; (vi) the CellAge database with >200 genes associated with cell senescence. All our databases are manually curated by experts and regularly updated to ensure a high quality data. Cross-links across our databases and to external resources help researchers locate and integrate relevant information. HAGR is freely available online (http://genomics.senescence.info/).

## INTRODUCTION

Ageing is a complex biological process that, despite decades of research, is not yet well understood. Many age-related changes have been described, however the theories regarding which mechanisms drive ageing changes are still controversial (1). Since their conception, the Human Ageing Genomic Resources (HAGR) have aimed to tackle this complex problem, rapidly becoming a leading online resource for biogerontologists. With the advent of large scale sequencing and breakthroughs in the genetics of ageing, HAGR has a particular (but not exclusive) focus on genomics.

As the field of ageing research has grown the amount of data being generated has rapidly increased. Since its first publication in 2005 (2), HAGR has expanded considerably to match this increase. Having started with only two databases, GenAge, a database of genes potentially associated with human ageing, and AnAge, a database of ageing and longevity in animals (2), HAGR now consists of six databases and a wide range of tools and resources tackling different aspects of ageing.

This article provides a non-technical description of the various databases, tools and projects in HAGR and their research applications. New resources created since the 2013 publication (3) are highlighted alongside updates to the remaining resources. In doing so we hope to provide a guide to HAGR so they can remain the most accessible and in-depth resources available online in the field of biogerontology. HAGR is freely available online (with no registration required) at http://genomics.senescence.info/.

## DATABASE CONTENT

### GenAge—the ageing gene database

The GenAge database (http://genomics.senescence.info/genes/) is the benchmark database of genes related to ageing. Since its first publication in 2005 (2), GenAge has progressed considerably (Table 1). At first, GenAge only included human genes potentially associated with ageing. Now the database is divided into two main sections: human potential ageing-associated genes and longevity-associated genes in model organisms. When the first HAGR paper was published in 2005 (2), GenAge contained 220 entries for human genes. Presently, build 19 (24/06/2017) of GenAge contains 307 human gene entries and 2152 entries for model organisms.

**Table 1.** Growth over time of the various datasets in the GenAge database

Database	Species	Number of gene entries			
		2005	2009	2013	2017
GenAge—Human genes	*Homo sapiens*	220	261	288	307
GenAge—Model organisms	*Mus musculus*		68	91	136
	*Caenorhabditis elegans*		555	680	877
	*Drosophila melanogaster*		75	128	193
	*Saccharomyces cerevisiae*		87	809	909
	**Total for model organisms**		785	1708	2115

GenAge—human genes (http://genomics.senescence.info/genes/human.html) contains a selection of genes which might affect the human ageing process. The focus is on genes implicated in multiple processes and pathologies related to ageing, so those genes affecting only a single age-related disease are excluded. Each gene in the dataset is annotated to indicate how it has been linked to human ageing and why it has been selected for inclusion in the database. The strongest level of evidence is for those genes directly linked to human ageing, typically those resulting in progeroid syndromes when mutated. Since our previous publication in 2013 (3), in addition to new gene entries, older gene entries have been updated to reflect additional findings from new publications. Currently >2500 publications are cited. Also included is a list of 73 genes whose expression is commonly altered during mammalian ageing (4).

**Figure 1.**
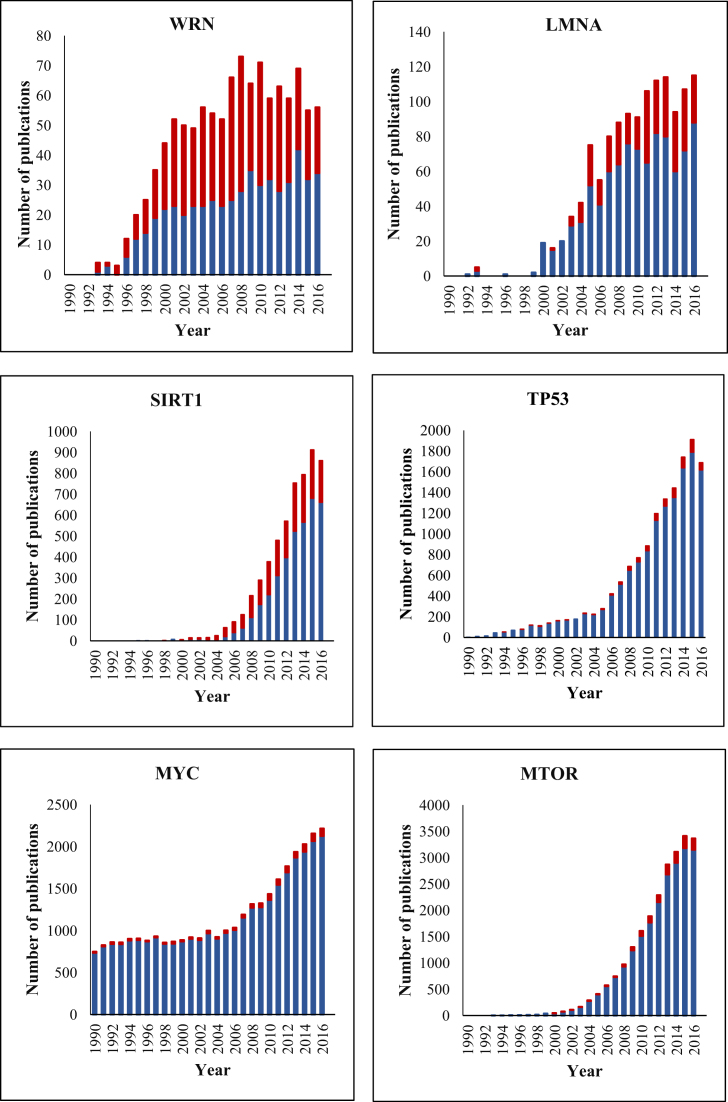
Patterns of ageing research for different human genes over time. The proportion of publications that focused mainly on ageing research are shown in red, the remaining publications are shown in blue.

Using data from GenAge—human genes we tracked patterns of ageing research over time. Research into specific genes in the context of ageing mostly began in the 1990s. Certain genes have become well-known through their role in ageing. Examples of these include *WRN*, the mutation of which results in Werner syndrome, possibly the most dramatic progeroid syndrome (5), *LMNA*, the mutation of which leads to Hutchinson-Gilford’s progeroid syndrome (6), and *SIRT1*, linked to several processes involved in ageing (7) (Figure 1). For well-studied genes, like *TP53*, an additional role in the ageing process has emerged over time. Examples of these include *MYC*, an oncogene mainly studied in the context of cancer (8), *MTOR*, a regulator of several cellular processes which was found to play a role in ageing in various model organisms (9), and *TP53*, a well-known tumour suppressor (10) (Figure 1).

GenAge—model organisms (http://genomics.senescence.info/genes/models.html) is a database of genes in model organisms which, if genetically modulated, result in significant changes in the ageing phenotype (e.g. progeroid syndromes in mice) and/or longevity (4). Most observations are from the four most popular biomedical model organisms: mice, worms, fruit flies and yeast (Table 1); however, several results from other model organisms such as zebrafish and golden hamsters are also included. For experiments using transgenic organisms, entries are classified according to the species in which the experiments were conducted in, not the species source of DNA. Where reported, the effects of the genetic manipulation on mean and/or maximal lifespan are included to provide quantitative data. As previously detailed (3), most genes are categorized as either pro- or anti-longevity depending on their effects on longevity; where studies report conflicting results, the genes are annotated as ‘unclear’. The size of the model organisms’ dataset has increased almost 3-fold since its conception in 2009, with over 400 genes added in the current update (Table 1), including miRNAs for the first time.

GenAge has proven a valuable resource for ageing research, as evidence by many publications. A systems level analysis of the GenAge human genes database identified a robust group of ageing-specific network characteristics, revealing ageing genes as network hubs (11). Moreover, in an analysis of genes in the ageing human brain, 54 genes with sustained, consistent expression and 23 genes with DNA methylation changes were found in GenAge (12). GenAge was also used to validate the targets of a serum miRNA profile of human longevity (13). The data from GenAge has been incorporated into other databases, including AgeFactDB (http://agefactdb.jenage.de/) (14) and the NetAge database (http://netage-project.org) (15). Therefore, although other databases with ageing-related genes exist, GenAge is the benchmark in the field.

### AnAge—the database of animal ageing and longevity

Comparative biology is an essential and growing approach in the biology of ageing (16). AnAge (http://genomics.senescence.info/species/) is an integrative database of longevity records for over 4000 organisms. It includes, if available, maximum longevity, taxonomy, metabolic characteristics, development schedules and a multitude of additional life history data. AnAge is now over a decade old (2), becoming the most widely used resource in HAGR (see below). Although other datasets with longevity data exist (17), AnAge is arguably the ‘gold standard’ for longevity data in animals given its regular updating and quality data from manual curation. Build 14 (14 October 2017) contains 4244 entries, mostly individual species but also entries for higher taxa like primates and mammals.

AnAge has previously been described in depth (3,18), and so its utility will only be briefly described. Entries contain maximum longevity and, where available, mortality parameters. Entries indicate whether the maximum longevity value comes from specimens kept in captivity or from the wild. Each entry includes a qualifier of confidence in the data and an estimate of sample size (3,18). Anecdotal evidence is not used to estimate maximum longevity but may be included in the observations. Factors that might introduce bias into comparative ageing studies, such as body size, metabolic rate, and development schedules, are also included where available (3,4,18). A list of species with negligible senescence is also provided.

The primary goal of AnAge is as a data source for comparative and evolutionary biogerontological studies, thus enabling researchers to study what factors influence differences in phenotype and longevity across phylogeny. For example, one large-scale study using data from AnAge investigated how multiple ecological and mode-of-life traits affect lifespan (19). The data from AnAge have also been incorporated into the Comparative Cellular and Molecular Biology of Longevity Database (http://genomics.brocku.ca/ccmbl/) (20), the MitoAge database (http://www.mitoage.info) (21), the Encyclopedia of Life (http://eol.org/), and the Animal Diversity Web (http://animaldiversity.ummz.umich.edu), demonstrating the versatility of this resource.

Recent updates for AnAge have mostly been qualitative. The rate of new species added has reduced over time—we have added 40 new species since the last HAGR publication (3)—but older entries are kept up to date with new findings in the field. Our latest update, build 14, included ∼150 new references. As evidence of the substantial curation efforts in AnAge, the observations in AnAge now total >50 000 words. While the main focus of AnAge remains on data in animals, particularly chordates, the database contains entries for traditional biomedical models, including invertebrates and fungi.

### GenDR—a database of dietary restriction-related genes

Dietary restriction (DR) delays the ageing process and extends lifespan in a multitude of species from yeast to mammals (22). However, the exact mechanisms of how DR extends lifespan are still unknown. As previously described (23), GenDR (http://genomics.senescence.info/diet/) is a database of DR-related genes. Herein, the use and function of GenDR will be briefly outlined along with updates since the 2013 HAGR paper (3).

DR-essential genes are defined in GenDR as those which, if genetically modified, interfere with DR-mediated lifespan extension (3,23). GenDR has entries for nematodes, fruit flies, mice, budding yeast and fission yeast. We recently (24 June 2017) released a new build of GenDR, which contains 214 DR-essential genes, a 35% increase (56 new genes) since our previous update (3). GenDR also contains a complimentary dataset of 173 genes consistently differentially expressed in mammals under DR (24).

GenDR is the first and, to our knowledge, only database of DR genes. We hope that GenDR may aid in the development of pharmacological DR mimetics. Indeed, GenDR was used to validate the gene targets of candidate DR mimetics in worms (25). In an analysis of the downstream targets of *daf-16*, a gene involved in DR in worms, four of the targets overlapped with the GenDR database, demonstrating the involvement of different components of the pathway in DR (26).

### LongevityMap—human genetic variants associated with longevity

Variation in human lifespan has been found to be 20–30% heritable, with increasing heritability at advanced ages (27). As next-generation sequencing and genome-wide approaches advance, so does the capacity for performing longevity association studies. To catalog the increasing volume of data in genetic studies of human longevity, we created LongevityMap (http://genomics.senescence.info/longevity/), a database of genes, gene variants and chromosomal locations associated with longevity (28). This differs from the GenAge database, which focuses mostly on data from model organisms and the few genes associated with human ageing (e.g. genes causing progeroid syndromes).

Entries in LongevityMap were compiled from the literature (28). Negative results are included to provide information regarding each gene and variant previously studied in the context of human longevity. Both large and small-scale studies are included, along with several cross-sectional studies and studies of extreme human longevity (e.g., in centenarians). Due to the diversity of data, details about the study design are outlined for each entry, such as population and sample size (28). Build 3 (24 June 2017) of LongevityMap contains 550 entries (a 9% increase in this latest update), 884 genes (18% increase) and 3144 variants (58% increase). Of the 550 entries, 275 are reported as significant findings. We hope that LongevityMap will act as a reference to help researchers parse the increasing quantities of data related to the genetics of human longevity.

### DrugAge—a database of ageing-related drugs

Identifying drugs that could extend lifespan in model organisms has received considerable interest (29). Our new DrugAge database (http://genomics.senescence.info/drugs/) is a curated database of drugs, compounds and supplements with anti-ageing effects that extend longevity in model organisms. Although another database of candidate geroprotectors exists, called Geroprotectors.org (30), DrugAge provides a more comprehensive and systematic dataset of life-extending drugs and compounds (31).

DrugAge was developed to allow researchers to prioritize drugs and compounds relevant to ageing, providing high-quality summary data in model organisms. As described (31), the data were primarily compiled from the literature, in addition to other databases and submissions from the scientific community. Build 2 (01 September 2016) of DrugAge contains 418 distinct compounds across 1316 lifespan assays on 27 unique model organisms.

Hundreds of genes in several pathways act as regulators of ageing (1,32). However, analysis of DrugAge and other HAGR databases has revealed that the overlap between the targets of lifespan-extending drugs and known ageing related genes is modest (31). This indicates that most ageing-related pathways have yet to be targeted pharmacologically; DrugAge may aid in guiding further assays. This was recently demonstrated in one study where machine learning was used to predict whether a compound would increase lifespan in worms using data from DrugAge. The best model had 80% prediction accuracy and the top hit compounds could broadly be divided into compounds affecting mitochondria, inflammation, cancer, and gonadotropin-releasing hormone (33).

### CellAge—a database of cell senescence genes

Cell senescence, also known as cellular senescence (CS), is the irreversible cessation of cell division of normally proliferating cells. Senescent cells accumulate as an organism ages and may be an important contributor to ageing and age-related disease (34). However, the connection between organismal ageing and CS remains controversial (35). CellAge (http://genomics.senescence.info/cells/) is a new database of CS-associated genes, built to elucidate mechanisms of CS and its role in ageing. It is described here for the first time.

To develop CellAge, a list of CS-associated genes was manually curated from the literature. Selection was based on gene manipulation experiments in human cells, which caused cells to induce or inhibit CS. The type of CS (replicative, stress-induced, or oncogene-induced), cell line, cell type and manipulation methods were standardized and recorded, facilitating the search and grouping of records of interest. The database includes data from primary cells in addition to immortalized cell lines and cancer cell lines. Each record contains observations about the evidence. Where reported, common markers of CS (36) such as growth arrest, increased SA-β-galactosidase activity, SA-heterochromatin foci, a decrease in BrdU incorporation, changes in morphology and specific gene expression signatures are described.

A Human Cellular Senescence Gene Database (HCSGD) has been recently described by others (37), yet it combines information from many distinct sources and types of evidence, while CellAge has a more clear and rigorous selection procedure as well as manual curation. The first build of CellAge contains 279 entries, in which experiments in lung fibroblasts, embryonic kidney cells and foreskin fibroblasts are the most widely represented in the data. The majority of genes are associated with replicative senescence (232 genes), followed by stress-induced senescence (34 genes) and oncogene-induced senescence (28 genes).

It is hoped that CellAge will aid in understanding the various types of CS and that analysis of the data will lead to the discovery of further CS-associated genes and their regulatory mechanisms. Analysis of the CellAge dataset is currently being carried out by our group and will be published in a future publication.

## TOOLS, PROJECTS AND OTHER INFORMATION RESOURCES

### Ageing-related disease genes

In industrialized societies, ageing is the main risk factor for many debilitating and life-threatening diseases including cancer, cardiovascular disease, arthritis, diabetes and neurodegeneration. As lifespan increases so too does the prevalence of these diseases (38). An understanding of how these diseases are linked to the ageing process is needed to help tackle this growing problem (39). Our new ageing-related disease genes tool (http://genomics.senescence.info/diseases/), first described here, makes available a set of age-related disease genes and permits their integration with ageing-related genes from our other databases.

The genes were assembled using data compiled by a National Institute of Ageing study (40), as described (41). Diseases with fewer than 20 genes associated were excluded from the gene list to avoid the dilution of findings. Processes and conditions such as insulin resistance and hyperlipidaemia were classified as dysfunctions and excluded from the list. Users can browse genes and diseases by MeSH disease terms, MeSH disease class and by gene symbol. The disease classes are cardiovascular diseases, immune system diseases, musculoskeletal diseases, neoplasms, nervous system diseases, and nutritional and metabolic diseases. Results can be grouped by gene or disease. There are 769 genes associated with 20 age-related diseases in total.

Our tool was designed so that age-related disease genes can be viewed, analyzed and downloaded in the context of ageing genes to understand potential functional overlap. The tool allows users to create a merged data set between age-related disease genes and ageing genes, according to user-defined filters. Where applicable, genes in HAGR databases can be converted into human homologs before merging.

### Cross-links and complementary resources

All our databases are fully integrated, allowing users to gain a deeper understanding of the genes and pathways involved in ageing. In particular genetic databases have extensive crosslinks between them, linking each entry in a database to entries in other databases where available. DrugAge is also integrated with other HAGR databases using drug-gene interaction data from DGIdb (42).

Moreover, for a greater understanding of ageing there are several additional resources to HAGR. Succinctly, the Digital Ageing Atlas (DAA, http://ageing-map.org/) is a database of human age-related changes at different biological levels (43). HAGR links to the DAA on its homepage and searches within HAGR also show results from the DAA where available. The Ageing Research Computational Tools (http://genomics.senescence.info/software/) are a toolkit of Perl modules aimed at parsing files, data-mining, and searching and downloading data from the Internet (2,4). An SPSS script is also available, which can be used to determine the demographic rate of ageing for a given population (44).

Senescence.info (http://www.senescence.info) is an informational repository on the science of ageing which aims to highlight the importance of ageing research and give an overview of current knowledge on the biology and genetics of ageing. Unlike HAGR, senescence.info is developed by a single person (J.P.M). Also in a informational and educational context, the WhosAge database (http://whoswho.senescence.info/) is a non-exhaustive list of individuals and companies working on ageing and longevity science, featuring 26 companies and 291 researchers.

Lastly, since the last update on HAGR (3), two social media resources have been made available on Facebook (https://www.facebook.com/pg/BiologyAgingNews) and Twitter (https://twitter.com/AgingBiology) which report on the latest news and findings in the field. The updates detail research in longevity, life-extension and rejuvenation technologies and link to articles/papers for further reading. These resources usually post several times a week to >6000 followers.

## DOWNLOADS AND AVAILABILITY

Our access policy remains the same as in our previous publications (2,3). All HAGR databases and resources are freely available at http://genomics.senescence.info/. All databases allow users the opportunity to export, download and reuse data for their own analyses, under a Creative Commons Attribution licence. Of note, for data from model organisms in GenAge and GenDR, users can not only download genes from each model organism but also homologs from other model organisms for each dataset. Lists of human homologs for all the genes from model organisms are also available. Feedback from users and colleagues is welcome and encouraged via email.

## DISCUSSION

Over the last decade, HAGR has expanded to include several new databases, datasets, tools and additional resources. Specifically, compared to our previous HAGR update (3), HAGR now includes the LongevityMap, DrugAge and CellAge databases. The older databases—GenAge, AnAge and GenDR—have been updated and enhanced with significant information and data. Overall, the databases in HAGR organize large quantities of complex data, putting the findings into context and aiding further analysis. Having organized databases is necessary for employing computational approaches to ageing (45), including machine learning (46) and systems biology approaches (47).

HAGR emphasizes high quality data on ageing and our databases are under continuous curation by experts in the field. AnAge provides information on data quality and sample size and prioritizes the reliability of the data over the most extreme values. GenAge—Model organisms, GenDR and CellAge all focus on genes from genetic manipulation experiments to ensure the selection process is as unbiased as possible. Nonetheless, some subjectivity is unavoidable and conflicting results can emerge. To cope with this, our policy is to be inclusive, providing evidence and links to the relevant literature and thus providing a balanced and comprehensive overview to the reader.

**Figure 2.**
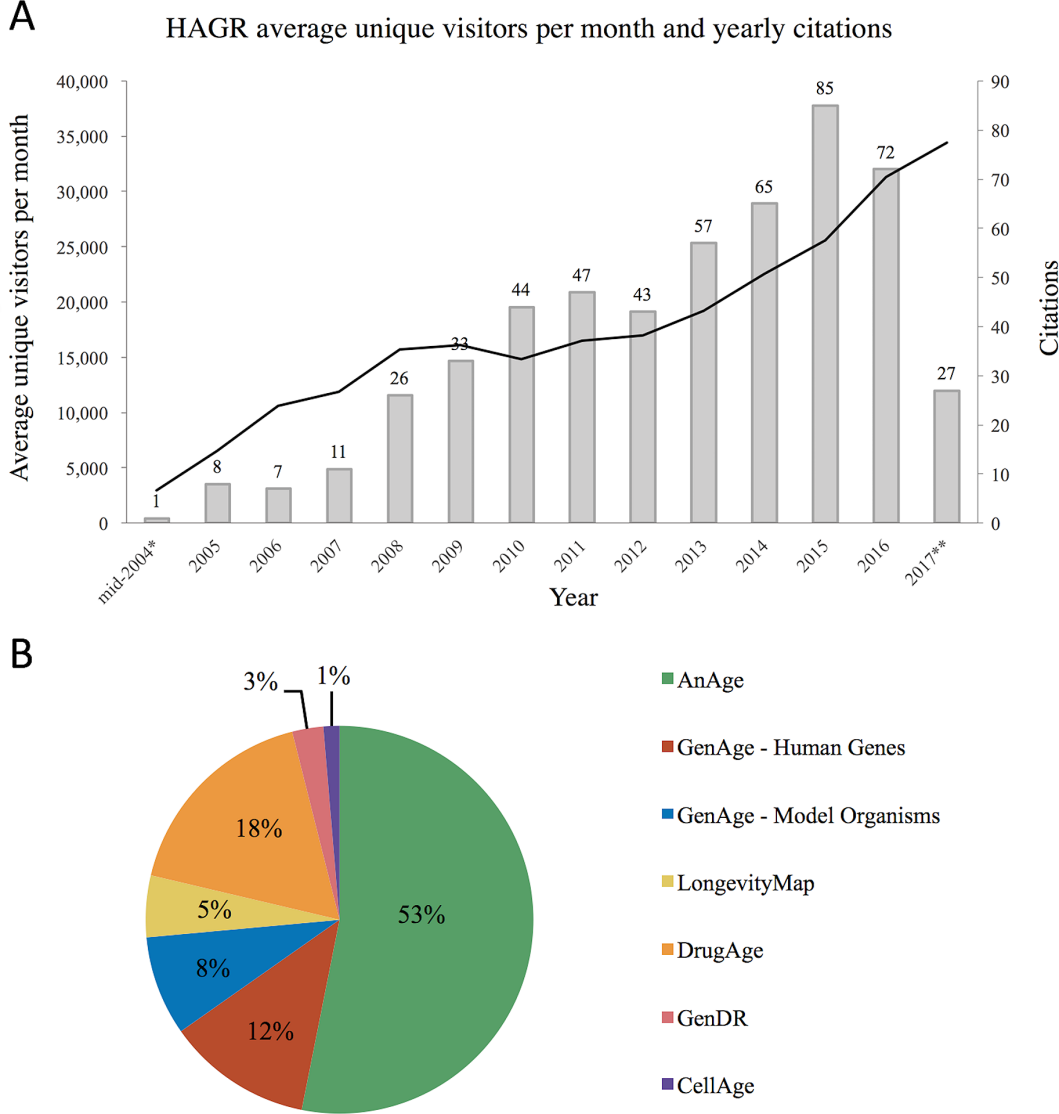
(**A**) Combined HAGR and senescence.info unique visitors per month (black line). The secondary axis and grey bars show the yearly growth in HAGR citations *HAGR became publicly available online in mid-2004. **Data to end of August 2017. (**B**) Usage by percentage of the different HAGR databases in 2017.

HAGR has been cited over 500 times since it was first published in 2005 and has seen a continuous rise in the number of citations over recent years. From 2006 our resources received over 10 000 unique visitors per month, and they now receive over 30 000 unique visitors per month, thus indicating HAGR’s growing importance in the field (Figure 2A). Out of all the HAGR databases, AnAge is the most popular (Figure 2B). GenAge—Human genes and GenAge—Model Organisms have collectively also maintained high levels of use. Since DrugAge was released in 2016 its usage has greatly increased, becoming one of the most widely used databases. CellAge is the newest HAGR resource, released in late 2016, hence not surprisingly still one of the least popular.

In conclusion, HAGR covers many aspects of ageing, acting as a science of ageing portal aimed at an audience from beginners to experts in biogerontology. Visitors are encouraged to send feedback and propose enhancements/features they would like to see in future. Over time as data continues to be generated, we anticipate that HAGR will continue to grow to meet this influx, maintaining its status as a leading online resource for studying the biology and genetics of ageing.

## References

[1] Lopez-Otin C., Blasco M.A., Partridge L., Serrano M., Kroemer G. The hallmarks of aging. Cell. 2013; 153:1194–1217. 23746838 10.1016/j.cell.2013.05.039 PMC3836174

[2] de Magalhaes J.P., Costa J., Toussaint O. HAGR: the Human Ageing Genomic Resources. Nucleic Acids Res. 2005; 33:D537–D543. 15608256 10.1093/nar/gki017 PMC539971

[3] Tacutu R., Craig T., Budovsky A., Wuttke D., Lehmann G., Taranukha D., Costa J., Fraifeld V.E., de Magalhaes J.P. Human Ageing Genomic Resources: integrated databases and tools for the biology and genetics of ageing. Nucleic Acids Res. 2013; 41:D1027–D033. 23193293 10.1093/nar/gks1155 PMC3531213

[4] de Magalhaes J.P., Budovsky A., Lehmann G., Costa J., Li Y., Fraifeld V., Church G.M. The Human Ageing Genomic Resources: online databases and tools for biogerontologists. Aging Cell. 2009; 8:65–72. 18986374 10.1111/j.1474-9726.2008.00442.x PMC2635494

[5] Goto M. Hierarchical deterioration of body systems in Werner's syndrome: implications for normal ageing. Mech. Ageing Dev. 1997; 98:239–254. 9352493 10.1016/s0047-6374(97)00111-5

[6] Eriksson M., Brown W.T., Gordon L.B., Glynn M.W., Singer J., Scott L., Erdos M.R., Robbins C.M., Moses T.Y., Berglund P. Recurrent de novo point mutations in lamin a cause Hutchinson-Gilford progeria syndrome. Nature. 2003; 423:293–298. 12714972 10.1038/nature01629 PMC10540076

[7] Bordone L., Guarente L. Calorie restriction, SIRT1 and metabolism: understanding longevity. Nat. Rev. Mol. Cell Biol. 2005; 6:298–305. 15768047 10.1038/nrm1616

[8] Henriksson M., Luscher B. Proteins of the Myc network: essential regulators of cell growth and differentiation. Adv. Cancer Res. 1996; 68:109–182. 8712067 10.1016/s0065-230x(08)60353-x

[9] Johnson S.C., Rabinovitch P.S., Kaeberlein M. mTOR is a key modulator of ageing and age-related disease. Nature. 2013; 493:338–345. 23325216 10.1038/nature11861 PMC3687363

[10] Ko L.J., Prives C. p53: puzzle and paradigm. Genes Dev. 1996; 10:1054–1072. 8654922 10.1101/gad.10.9.1054

[11] Zhang Q., Nogales-Cadenas R., Lin J.R., Zhang W., Cai Y., Vijg J., Zhang Z.D. Systems-level analysis of human aging genes shed new light on mechanisms of aging. Hum. Mol. Genet. 2016; 25:2934–2947. 27179790 10.1093/hmg/ddw145 PMC6390412

[12] Meng G., Zhong X., Mei H. A systematic investigation into aging related genes in brain and their relationship with Alzheimer's disease. PLoS One. 2016; 11:e0150624. 26937969 10.1371/journal.pone.0150624 PMC4777381

[13] Smith-Vikos T., Liu Z., Parsons C., Gorospe M., Ferrucci L., Gill T.M., Slack F.J. A serum miRNA profile of human longevity: findings from the Baltimore Longitudinal Study of Aging (BLSA). Aging (Albany NY). 2016; 8:2971–2987. 27824314 10.18632/aging.101106 PMC5191881

[14] Huhne R., Thalheim T., Suhnel J. AgeFactDB–the JenAge Ageing Factor Database–towards data integration in ageing research. Nucleic Acids Res. 2014; 42:D892–D896. 24217911 10.1093/nar/gkt1073 PMC3964983

[15] Tacutu R., Budovsky A., Fraifeld V.E. The NetAge database: a compendium of networks for longevity, age-related diseases and associated processes. Biogerontology. 2010; 11:513–522. 20186480 10.1007/s10522-010-9265-8

[16] Austad S.N. Cats, “rats,” and bats: the comparative biology of aging in the 21st century. Integr. Comp. Biol. 2010; 50:783–792. 21558241 10.1093/icb/icq131 PMC3140272

[17] Carey J.R., Judge D.S. Cats, “Rats,” and Bats: the Comparative Biology of Aging in the 21st Century. 2000; Odense: Odense University Press.

[18] de Magalhaes J.P., Costa J. A database of vertebrate longevity records and their relation to other life-history traits. J. Evol. Biol. 2009; 22:1770–1774. 19522730 10.1111/j.1420-9101.2009.01783.x

[19] Healy K., Guillerme T., Finlay S., Kane A., Kelly S.B., McClean D., Kelly D.J., Donohue I., Jackson A.L., Cooper N. Ecology and mode-of-life explain lifespan variation in birds and mammals. Proc. Biol. Scie. 2014; 281:20140298. 10.1098/rspb.2014.0298 PMC4043093 24741018

[20] Stuart J.A., Liang P., Luo X., Page M.M., Gallagher E.J., Christoff C.A., Robb E.L. A comparative cellular and molecular biology of longevity database. Age (Dordr). 2013; 35:1937–1947. 22836712 10.1007/s11357-012-9458-y PMC3776122

[21] Toren D., Barzilay T., Tacutu R., Lehmann G., Muradian K.K., Fraifeld V.E. MitoAge: a database for comparative analysis of mitochondrial DNA, with a special focus on animal longevity. Nucleic Acids Res. 2016; 44:D1262–D1265. 26590258 10.1093/nar/gkv1187 PMC4702847

[22] Fontana L., Partridge L., Longo V.D. Extending healthy life span–from yeast to humans. Science. 2010; 328:321–326. 20395504 10.1126/science.1172539 PMC3607354

[23] Wuttke D., Connor R., Vora C., Craig T., Li Y., Wood S., Vasieva O., Shmookler Reis R., Tang F., de Magalhaes J.P. Dissecting the gene network of dietary restriction to identify evolutionarily conserved pathways and new functional genes. PLoS Genet. 2012; 8:e1002834. 22912585 10.1371/journal.pgen.1002834 PMC3415404

[24] Plank M., Wuttke D., van Dam S., Clarke S.A., de Magalhaes J.P. A meta-analysis of caloric restriction gene expression profiles to infer common signatures and regulatory mechanisms. Mol. Biosyst. 2012; 8:1339–1349. 22327899 10.1039/c2mb05255e

[25] Calvert S., Tacutu R., Sharifi S., Teixeira R., Ghosh P., de Magalhaes J.P. A network pharmacology approach reveals new candidate caloric restriction mimetics in C. elegans. Aging Cell. 2016; 15:256–266. 26676933 10.1111/acel.12432 PMC4783339

[26] Li Y.H., Zhang G.G. Towards understanding the lifespan extension by reduced insulin signaling: bioinformatics analysis of DAF-16/FOXO direct targets in Caenorhabditis elegans. Oncotarget. 2016; 7:19185–19192. 27027346 10.18632/oncotarget.8313 PMC4991374

[27] Christensen K., Johnson T.E., Vaupel J.W. The quest for genetic determinants of human longevity: challenges and insights. Nat. Rev. Genet. 2006; 7:436–448. 16708071 10.1038/nrg1871 PMC2726954

[28] Budovsky A., Craig T., Wang J., Tacutu R., Csordas A., Lourenco J., Fraifeld V.E., de Magalhaes J.P. LongevityMap: a database of human genetic variants associated with longevity. Trends Genet. 2013; 29:559–560. 23998809 10.1016/j.tig.2013.08.003

[29] de Magalhaes J.P., Stevens M., Thornton D. The business of anti-aging science. Trends Biotechnol. 2017; 35:1062–1073. 28778607 10.1016/j.tibtech.2017.07.004

[30] Moskalev A., Chernyagina E., de Magalhaes J.P., Barardo D., Thoppil H., Shaposhnikov M., Budovsky A., Fraifeld V.E., Garazha A., Tsvetkov V. Geroprotectors.org: a new, structured and curated database of current therapeutic interventions in aging and age-related disease. Aging (Albany NY). 2015; 7:616–628. 26342919 10.18632/aging.100799 PMC4600621

[31] Barardo D., Thornton D., Thoppil H., Walsh M., Sharifi S., Ferreira S., Anzic A., Fernandes M., Monteiro P., Grum T. The DrugAge database of aging-related drugs. Aging Cell. 2017; 16:594–597. 28299908 10.1111/acel.12585 PMC5418190

[32] de Magalhaes J.P., Wuttke D., Wood S.H., Plank M., Vora C. Genome-environment interactions that modulate aging: powerful targets for drug discovery. Pharmacol. Rev. 2012; 64:88–101. 22090473 10.1124/pr.110.004499 PMC3250080

[33] Barardo D.G., Newby D., Thornton D., Ghafourian T., de Magalhaes J.P., Freitas A.A. Machine learning for predicting lifespan-extending chemical compounds. Aging (Albany NY). 2017; 9:1721–1737. 28783712 10.18632/aging.101264 PMC5559171

[34] Campisi J., d’Adda di Fagagna F. Cellular senescence: when bad things happen to good cells. Nat. Rev. Mol. Cell Biol. 2007; 8:729–740. 17667954 10.1038/nrm2233

[35] de Magalhaes J.P., Passos J.F. Stress, cell senescence and organismal ageing. Mech. Ageing Dev. 2017; doi:10.1016/j.mad.2017.07.001. 10.1016/j.mad.2017.07.001 28688962

[36] Carnero A. Markers of cellular senescence. Methods Mol. Biol. 2013; 965:63–81. 23296651 10.1007/978-1-62703-239-1_4

[37] Dong Q., Han H., Liu X., Wei L., Zhang W., Zhao Z., Zhang M.Q., Wang X. HCSGD: an integrated database of human cellular senescence genes. J. Genet. Genomics. 2017; 44:227–234. 28529078 10.1016/j.jgg.2017.04.001

[38] Niccoli T., Partridge L. Ageing as a risk factor for disease. Curr. Biol. 2012; 22:R741–R752. 22975005 10.1016/j.cub.2012.07.024

[39] Kennedy B.K., Berger S.L., Brunet A., Campisi J., Cuervo A.M., Epel E.S., Franceschi C., Lithgow G.J., Morimoto R.I., Pessin J.E. Geroscience: linking aging to chronic disease. Cell. 2014; 159:709–713. 25417146 10.1016/j.cell.2014.10.039 PMC4852871

[40] Zhang Y., De S., Garner J.R., Smith K., Wang S.A., Becker K.G. Systematic analysis, comparison, and integration of disease based human genetic association data and mouse genetic phenotypic information. BMC Med. Genomics. 2010; 3:1. 20092628 10.1186/1755-8794-3-1 PMC2822734

[41] Fernandes M., Wan C., Tacutu R., Barardo D., Rajput A., Wang J., Thoppil H., Thornton D., Yang C., Freitas A. Systematic analysis of the gerontome reveals links between aging and age-related diseases. Hum. Mol. Genet. 2016; 25:4804–4818. 28175300 10.1093/hmg/ddw307 PMC5418736

[42] Wagner A.H., Coffman A.C., Ainscough B.J., Spies N.C., Skidmore Z.L., Campbell K.M., Krysiak K., Pan D., McMichael J.F., Eldred J.M. DGIdb 2.0: mining clinically relevant drug-gene interactions. Nucleic Acids Res. 2016; 44:D1036–D1044. 26531824 10.1093/nar/gkv1165 PMC4702839

[43] Craig T., Smelick C., Tacutu R., Wuttke D., Wood S.H., Stanley H., Janssens G., Savitskaya E., Moskalev A., Arking R. The Digital Ageing Atlas: integrating the diversity of age-related changes into a unified resource. Nucleic Acids Res. 2015; 43:D873–D878. 25232097 10.1093/nar/gku843 PMC4384002

[44] de Magalhaes J.P., Cabral J.A., Magalhaes D. The influence of genes on the aging process of mice: a statistical assessment of the genetics of aging. Genetics. 2005; 169:265–274. 15466429 10.1534/genetics.104.032292 PMC1448866

[45] Wieser D., Papatheodorou I., Ziehm M., Thornton J.M. Computational biology for ageing. Philos. Trans. R. Soc. Lond. B. Biol. Sci. 2011; 366:51–63. 21115530 10.1098/rstb.2010.0286 PMC3001313

[46] Fabris F., Magalhaes J.P., Freitas A.A. A review of supervised machine learning applied to ageing research. Biogerontology. 2017; 18:171–188. 28265788 10.1007/s10522-017-9683-y PMC5350215

[47] Kriete A., Lechner M., Clearfield D., Bohmann D. Computational systems biology of aging. Wiley Interdiscip. Rev. Syst. Biol. Med. 2011; 3:414–428. 21197651 10.1002/wsbm.126

